# Nano-Fibrillated Bacterial Cellulose Nanofiber Surface Modification with EDTA for the Effective Removal of Heavy Metal Ions in Aqueous Solutions

**DOI:** 10.3390/ma18020374

**Published:** 2025-01-15

**Authors:** Sayaka Fujita, Ryosui Sasa, Nanami Kinoshita, Ryota Kishimoto, Hiroyuki Kono

**Affiliations:** 1 Division of Applied Chemistry and Biochemistry, National Institute of Technology, Tomakomai College, Nishikioka 443, Tomakomai 059-1275, Hokkaido, Japan; 2 Graduate School of Environmental Science, Hokkaido University, N10W5, Kita-ku, Sapporo 060-0810, Hokkaido, Japan

**Keywords:** cellulose nanofiber, bacterial cellulose, ethylenediaminetetraacetic acid, heavy metal adsorption, surface modification

## Abstract

Nano-fibrillated bacterial cellulose (NFBC) has very long fibers (>17 μm) with diameters of approximately 20 nm. Hence, they have a very high aspect ratio and surface area. The high specific surface area of NFBC can potentially be utilized as an adsorbent. However, NFBC has no functional groups that can bind metal ions, limiting its potential applications. In this study, the hydroxyl groups on the surface of NFBC were chemically modified with EDTA monoanhydride to convert NFBC into a metal adsorbent. The fiber morphology and crystal structures of the modified NFBC were almost identical to those of the unmodified NFBC, suggesting that the surface hydroxyl groups of NFBC were well-conjugated with the EDTA groups. Surface-modified NFBC preferentially adsorbed transition metals in aqueous solutions, such as Cu(II), Hg(II), Pb(II), and Cd(II), but hardly adsorbed Mg(II) and Cr(VI). The adsorption of heavy metal ions can be explained by the pseudo-second-order kinetics of the chemisorption process and the Langmuir isotherm model. Furthermore, the EDTA-modified NFBC is a renewable and recyclable adsorbent. The results of this study indicate that surface-modified NFBC can be utilized as a biosorbent for heavy metal removal in chemical, food, pharmaceutical, and other industrial fields.

## 1. Introduction

Heavy metals and their compounds are essential raw materials in many industrial processes, including electroplating, metallurgy, textiles, and numerous other sectors [1]. Heavy metals are highly toxic, non-biodegradable, and tend to readily accumulate in living organisms through the food chain. For example, lead, copper, and cadmium accumulate within organisms and cause cancer, anemia, hypertension, nervous system damage, and kidney failure, posing a significant threat to human health [2,3,4]. Pollutants, including heavy metals, will inevitably continue to be released into the environment through wastewater as long as human and industrial activities persist. Therefore, it is necessary to explore effective methods for treating heavy metal contaminants in wastewater to benefit human health and the environment.

The current technologies used to remove toxic heavy metal ions in wastewater include membrane separation, coagulation, flocculation, adsorption, ion exchange, chemical precipitation, phytoremediation, treatment with living microbes, and biofiltration [5,6,7]. However, several of these methods have limitations, such as the production of large amounts of sludge, the generation of secondary pollutants, incomplete pollutant removal, and high operational and maintenance costs. Therefore, to overcome these problems, it is imperative to develop low-cost, environmentally friendly, and effective wastewater treatment methods.

Adsorption methods are potentially cost-effective because of their high adsorption efficiency, ease of operation, and applicability to large-scale industrial production [8,9]. Crucial factors in the application of adsorbent materials include high adsorption capacity, regeneration feasibility, and ecological compatibility. Cellulose nanofibers (CNFs) are abundant, renewable, and sustainable biomass materials that possess properties such as biodegradability, non-toxicity, durability, and thermal and mechanical stability [10,11]. Regarding ecological compatibility, a crucial criterion for adsorbents, CNFs are suitable candidates owing to their derivation from cellulose. Moreover, the relatively large and specific surface area of CNFs facilitates the adsorption process as an adsorbent, as it allows for the formation of favorable contacts between the adsorption site and the adsorbate. The remarkable mechanical strength of CNFs, derived from its highly crystalline structure, ensures exceptional durability during repeated use and regeneration cycles. This property renders it an ideal candidate for long-term applications in industrial adsorption processes, surpassing other cellulose materials, such as microcrystalline cellulose, in terms of both durability and reusability. One type of CNF is nano-fibrillated bacterial cellulose (NFBC), which is prepared by culturing *Gluconacetobacter intermedium* NEDO-1 in an aqueous medium containing water-soluble cellulose derivatives such as carboxymethyl cellulose (CMC) and hydroxyethyl cellulose under aerobic conditions, with continuous stirring during the culture process [12,13]. Unlike the standard cultivation method for bacterial cellulose (BC), the water-soluble cellulose derivatives in the medium adsorb onto the surface of the extracellular BC microfibrils or their bundles produced by *Acetobacter*. This adsorption prevents the self-assembly of the microfibrils, resulting in the formation of nanofibers with a finer diameter of approximately 20 nm, which is thinner than that of standard BC. The preparation of CNFs typically involves the mechanical or chemical processing of wood, resulting in a mixture of microfibers with uneven fiber diameters and short fiber lengths. However, NFBC is produced by microorganisms, which circumvents the destruction of cellulose fiber bundles that may occur during mechanical or chemical treatments. Consequently, NFBC exhibits exceptionally long fiber lengths exceeding 17 μm, with uniform fiber diameters and no mixture of microfibers. This results in aspect ratios greater than 1000, more than 10 times higher than those of 2,2,6,6-tetramethylpioeridine-1-oxyl (TEMPO)-oxidized CNFs [14]. In summary, NFBC has excellent properties, such as a very high aspect ratio, large surface area, and the presence of homogeneous fibers. In contrast to CNFs derived from wood, which frequently necessitate energy-intensive mechanical or chemical processing, NFBC production relies on microbial synthesis, a more environmentally friendly process. This process utilizes renewable resources and does not necessitate the use of harsh chemicals, thereby aligning with the principles of green chemistry and sustainable material production.

**Figure 1:**
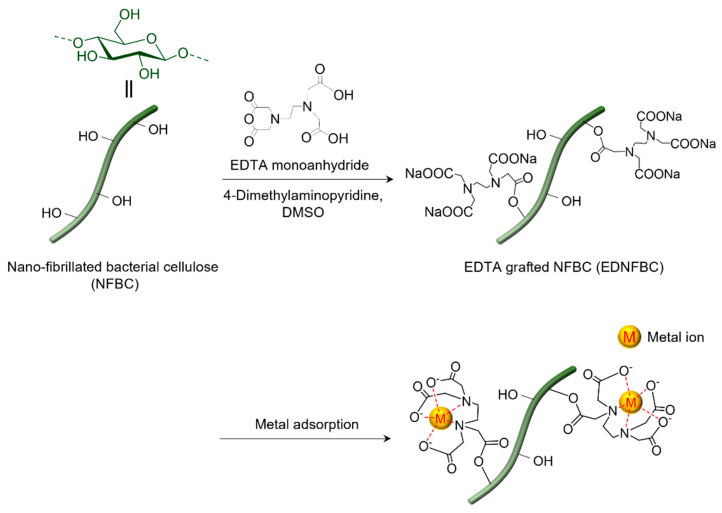
Preparation of EDTA grafted nano-fibrillated bacterial cellulose (EDNFBC) using EDTA monoanhydride (EDTAM).

In this study, we aimed to prepare chelated nanofibers with an extremely high aspect ratio and a super-specific surface area effect by substituting ethylenediaminetetraacetic acid (EDTA), a strong chelating agent, with hydroxyl groups on the fiber surface of NFBC (Figure 1). These unique properties are expected to distinguish NFBC from conventional cellulose-based materials, significantly enhancing its adsorption performance. EDTA can form chelates with most metal ions, regardless of their valence. While NFBC lacks functional groups to directly bind to metal ions, its numerous hydroxyl groups can be modified with EDTA, making it effective for metal ion removal. Several studies have explored materials modified with EDTA for similar applications. For example, cellulose filter paper modified with EDTA can remove various monovalent and divalent metal ions, demonstrating its potential as a filtration membrane [15]. In addition, mercerized cellulose derived from sugarcane bagasse or pineapple modified with EDTA exhibits excellent adsorption capacities for divalent metals [16,17]. Similarly, functionalizing commercially available cotton Aida cloth with EDTA can enhance its adsorption capacity for Ni(II) and its recyclability, providing a reusable adsorption fabric [18]. Overall, these studies indicate that EDTA dianhydrides are commonly used to functionalize cellulose materials [19,20]. However, the two reactive sites of EDTA dianhydride can often lead to extensive intermolecular crosslinking during the reaction [21,22]. This crosslinking can reduce the dispersibility of the resulting material in aqueous systems, potentially leading to the formation of viscous gels that are challenging to handle and may compromise adsorption performance. These limitations pose challenges for practical applications. To address these challenges, in this study, EDTA monoanhydride (EDTAM) was selected as the functionalizing agent. Unlike EDTA dianhydride, monoanhydride has only one reactive site, minimizing crosslinking reactions [23]. The synthesized EDTA-grafted NFBC (EDNFBC) was subjected to structural characterization via Fourier-transform infrared (FTIR) spectroscopy, X-ray diffraction (XRD), and thermogravimetry/differential thermal analysis (TG/DTA). Furthermore, the adsorption performance of EDNFBC for metal ions was evaluated. The impact of pH on the adsorption of metal ions onto EDNFBC was investigated, along with the kinetics and isotherms of the adsorption process. This analysis was performed to elucidate the adsorption mechanisms of NFBC. Moreover, the recycling efficiencies of the adsorbents were also determined.

## 2. Results and Discussion

### 2.1. Preparation of EDNFBC

The choice of NFBC as the base material was driven by its remarkably high specific surface area and nanoscale structure, which facilitate efficacious interactions with metal ions. The primary objective was to develop a material with an enhanced metal adsorption capacity by leveraging the surface area effect of NFBC while preserving its fibrous morphology. To this end, NFBC was functionalized with EDTAM to selectively introduce EDTA to the surface without disrupting the nanofiber structure. Specifically, the reaction was conducted by suspending NFBC in DMSO, followed by the addition of EDTAM and 4-dimethylaminopyridine (DMAP) as a basic catalyst (Figure 1). The preparation of EDNFBC samples is described in the Supporting Information. Briefly, commercially available NFBC dispersed in water (Kusano Sakko Inc., Ebetsu, Hokkaido, Japan, 1.0% (*w*/*v*)) was subjected to centrifugation to remove excess water. The concentrated NFBC slurry was subsequently resuspended in DMSO in preparation for the reaction. EDTAM and DMAP were added to the suspension, and the mixture was stirred at 298 K for 24 h. After the completion of the reaction, the product was separated through centrifugation, washed with deionized water, and dialyzed to remove unreacted reagents. EDNFBC with different degrees of EDTA substitution were achieved by adjusting the amount of EDTAM (Table 1).

### 2.2. Structural Characterization of EDNFBC

#### 2.2.1. FTIR Spectroscopy

**Figure 2:**
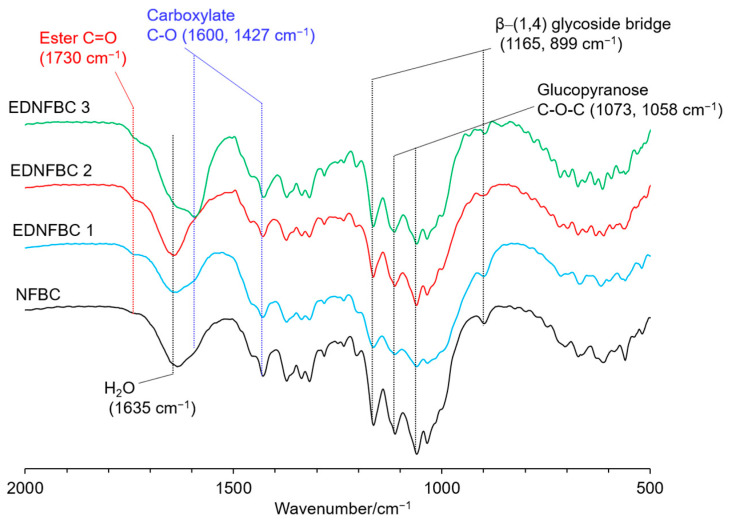
FTIR spectra of NFBC and EDNFBC 1–3.

Figure 2 shows the FTIR spectra of EDNFBC 1–3 and NFBC as starting materials. In these spectra, the absorption bands of the β–(1,4) glycoside bridge were detected at 1165 and 899 cm^−1^, along with the C–O–C stretching vibrations in glucopyranose at 1073 and 1058 cm^−1^ [24]. Since the glucopyranose in NFBC remained unaltered during the grafting reaction with the EDTA process to produce EDNFBC, the spectra were normalized using the absorption of glucopyranose at 1058 cm^−1^. The spectrum of NFBC displayed two adsorption bands at 1600 and 1460 cm^−1^, corresponding to the C–O asymmetrical and symmetrical stretching vibrations of the carboxylate group, respectively [25]. Because NFBC was prepared in a medium containing CMC, CMC attached to the surface of NFBC. These absorption bands at 1600 and 1460 cm^−1^ were attributed to the carboxylate groups in CMC. In comparison to NFBC, the EDNFBC spectra showed an additional absorption band at 1730 cm^−1^, which corresponded to the C=O stretching of the ester [26]. This implied that the carboxyl group of EDTA reacts with the hydroxyl groups of NFBC to form ester bonds. The intensity of the C=O band increased with an increase in the feed amount of EDTAM, suggesting that the amount of EDTA grafted onto the NFBC also increased. In addition, the absorption band at 1635 cm^−1^ was present in all spectra, which was due to the bending vibration of residual water molecules in the sample [27] and not due to the structure of NFBC or EDNFBC.

#### 2.2.2. XRD

**Figure 3:**
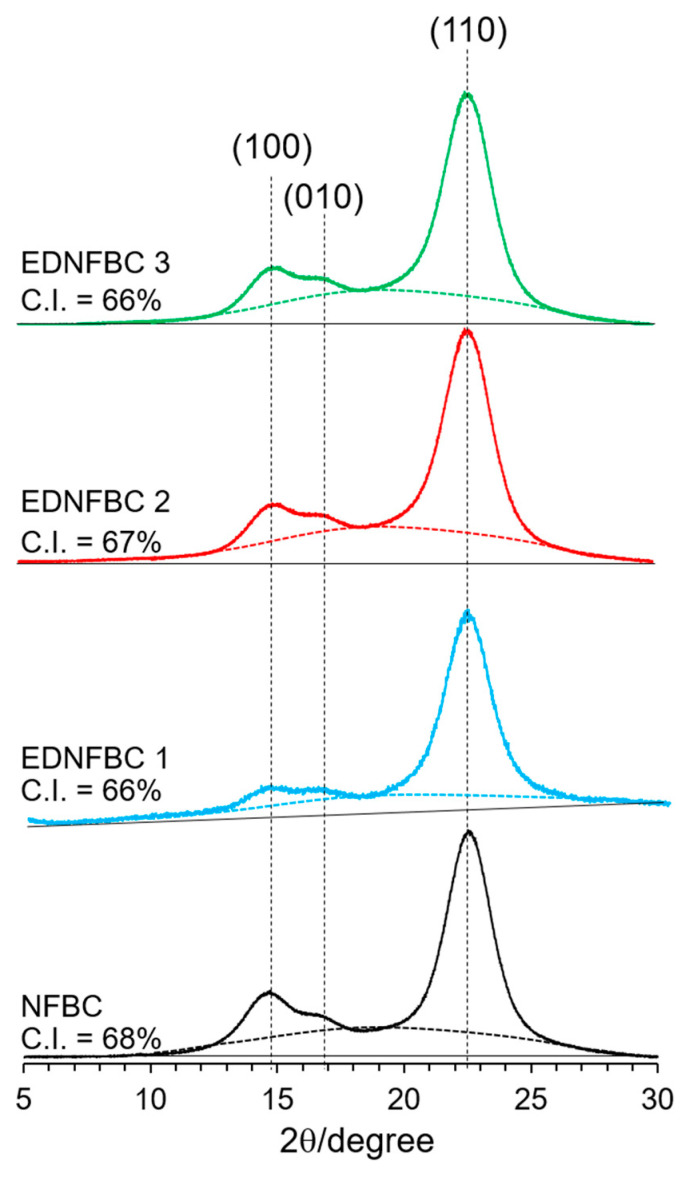
XRD patterns of NFBC and EDNFBC 1–3.

Figure 3 shows the X-ray patterns of NFBC and EDNFBC 1–3. All samples exhibited characteristic diffraction peaks at 14.4°, 16.8°, and 22.8°, corresponding to the (100), (010), and (110) crystal planes of cellulose Iα, respectively [28]. The crystallinity index (C.I.) was calculated by determining the ratio of the crystalline cellulose peak area to the total area under the diffractogram. To identify the amorphous regions, curve fitting was applied to the diffractograms with the amorphous areas represented below the dotted line in Figure 3. The amorphous content for NFBC and EDNFBC 1–3 were estimated to be 32, 34, 35, and 34% of the total areas, revealing minimal variations among the samples. The C.I. for NFBC and EDNFBC 1–3 were 68, 66, 65%, and 66%, respectively. As EDTA substitution occurs within the NFBC fibers, it was expected that the intermolecular hydrogen bonds in cellulose were cleaved, resulting in a reduction in crystallinity. The C.I. of EDNFBC was almost the same as that of NFBC, suggesting that the inner cellulose structure remained unchanged during the reaction of EDTA substitution and that EDTA substitution proceeded on the surface of the NFBC fibers.

### 2.3. Morphologies of EDNFBC

**Figure 4:**
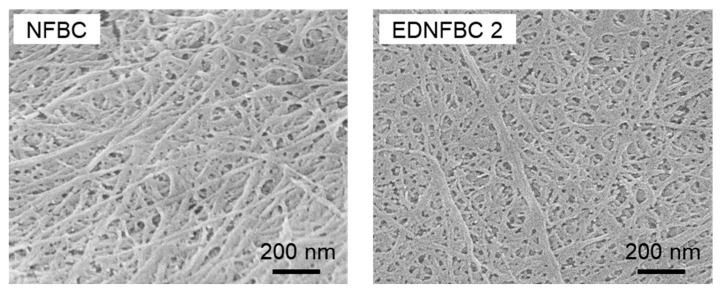
SEM images of NFBC and EDNFBC 2.

Figure 4 shows SEM images of NFBC and EDNFBC 2, observing their fine fibrillar structures. Fiber widths were measured for 50 randomly selected fibers from each sample. The average fiber width of NFBC was 23.4 ± 7.5 nm, while that of EDNFBC 2 was 23.6 ± 7.8 nm. These measurements indicated that the fiber width remained consistent before and after the substitution reaction with EDTA. This observation suggests that the EDTA modification occurred on the surface of the NFBC fibers without affecting their internal structure. This conclusion was consistent with the results of the XRD analysis, which also revealed that the EDTA substitution reaction occurred on the surface of the NFBC fibers with no discernible impact on the NFBC fibers.

### 2.4. Adsorption Properties of Metal Ions

#### 2.4.1. Effect of pH on Adsorption

The pH of the solution affects the protonation of the three carboxyl groups and two amines in EDTA and the speciation of metal ions in the solution. Therefore, the optimal pH must be determined to maximize metal ion removal. The effect of pH on adsorption was also examined using Cu(II) as the model metal ion pollutant. Cu(II) tends to form hydroxide precipitates at pH levels above 5.5, potentially distorting adsorption studies and affecting the results [29]. To ensure that the adsorption performance of EDNFBC was evaluated exclusively, without interference from hydroxide precipitation, the experiments in this study were conducted within a pH range of 2.0–5.0, where the effect of hydroxide precipitation was minimal. This pH range also aligned with the typical acidic conditions of industrial effluents containing Cu(II) where pH levels are often below five, making the findings relevant to practical applications. Adsorption experiments were performed with an initial Cu(II) concentration of 25 mg/L and an EDNFBC 2 dose of 0.9 mg/mL. The adsorption capacity (*q*) was calculated using Equation (1) as follows:

(1) $$q=\frac{(C_{0}-C_{f})V}{m}$$

where *C*_0_ (mg/L) is the initial Cu(II) concentration, *C_f_* is the final Cu (II) concentration, *V* (L) is the volume of the Cu(II) solution, and *m* (g) is the weight of EDNFBC.

**Figure 5:**
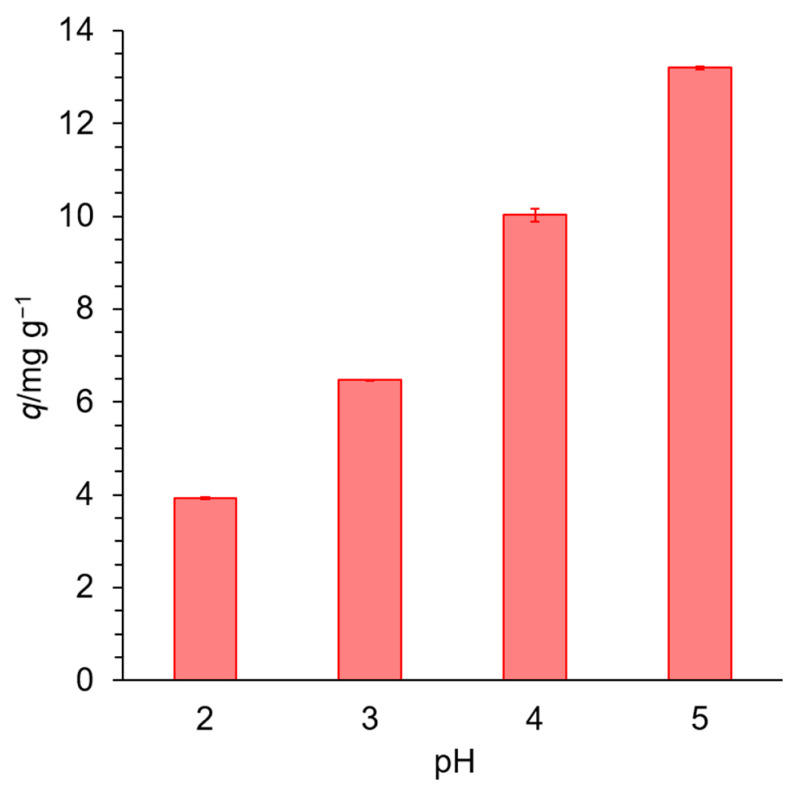
The effect of pH on the adsorption of Cu(II). The initial concentration of Cu(II) was 25 mg/L, the dosage of EDNFBC 2 was 0.9 mg/mL, the contact time was 120 min, the shaking rate was 120 rpm, and the temperature was 298 K.

Figure 5 shows the effect of pH on adsorption onto EDNFBC 2. The results show that *q* increased with increasing pH, indicating that adsorption was strongly dependent on the pH of the solution. The EDTA introduced into NFBC had two nitrogen atoms and three carboxylic acid groups in its structure. Chelation occurs through the coordination of Cu(II) with the oxygen atoms in the carboxylic acid groups and the lone electron pairs on the nitrogen atoms [30]. At low pH, the nitrogen atoms are protonated, and the carboxylic groups are almost undissociated, resulting in the EDTA groups decreasing their coordination ability with metal ions and EDNFBC exhibiting low adsorption. With increasing pH, deprotonation of the carboxylic groups occurs, resulting in increased coordination ability and, subsequently, high adsorption of EDNFBC.

#### 2.4.2. Adsorption Kinetics

**Figure 6:**
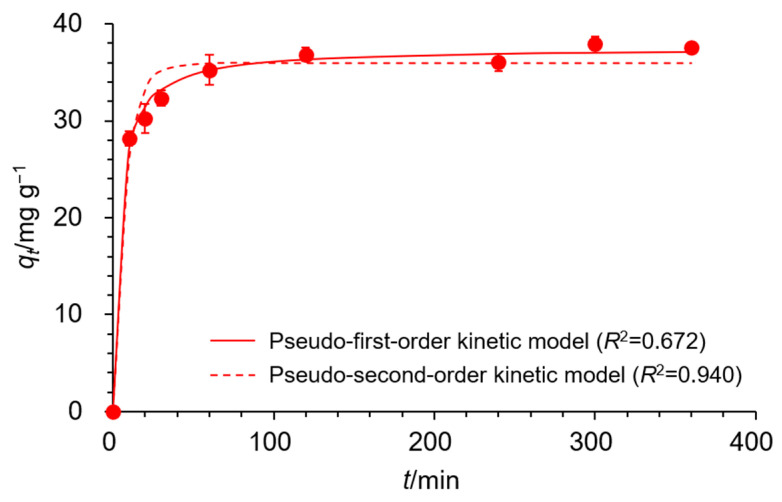
The effect of contact time on the adsorption of Cu(II) onto EDNFBC 2 as fitted to the pseudo-first- and pseudo-second-order kinetic models. The experiments were performed under the following conditions: EDNFBC dose, 0.9 mg/mL; saturation concentration of the adsorbate (*C*_0_), 100 mg/L; pH, 5; temperature, 298 K.

The contact time between the adsorbent and adsorbate is a critical factor in understanding the adsorption mechanism. Next, the effect of contact time on the adsorption of EDNFBC was investigated using Cu(II) (Figure 6). The adsorption capacity at the contact time t (*q_t_*) was calculated using Equation (2) as follows:

(2) $$q_{t}=\frac{(C_{0}-C_{t})V}{m}$$

where *C*_0_ (mg/L) is the initial concentration of Cu(II), *C_t_* is the concentration of Cu(II) at the contact time, *V* (L) is the volume of the Cu(II) solution, and *m* (g) is the weight of EDNFBC. The results revealed a rapid increase in adsorption within the initial period, which subsequently plateaued at 120 min, indicating that equilibrium was reached. To further investigate the kinetic behavior and rate-determining step of the adsorption process, the obtained data were fitted using three kinetic models: the pseudo-first-order, pseudo-second-order, and Elovich kinetic models. The pseudo-first-order kinetic model theorizes that adsorption is driven by free diffusion of the adsorbate [31] and is expressed in Equation (3) as follows:

(3) $$q_{t}=q_{e}(1-e^{{-k}_{1}t})$$

where *k*_1_ is the rate constant of the pseudo-first-order kinetic model, *q_e_* is the adsorption capacity of EDNFBC at equilibrium, *t* is the contact time, and *q_t_* is the adsorption capacity at each time interval.

The pseudo-second-order kinetic model presumes that chemisorption is the rate-controlling step, involving electron exchange or the formation of covalent bonds between the adsorbent and adsorbate [32]. The pseudo-second-order kinetic model is expressed in Equation (4) below:

(4) $$q_{t}=\frac{k_{2}{q_{e}}^{2}t}{1+k_{2}q_{e}t}$$

where *k*_2_ is the rate constant of the pseudo-second-order kinetics model.

Finally, the Elovich kinetic model was used to describe diverse processes, including surface diffusion, bulk diffusion, and chemisorption. This model allowed for the establishment of a process based on diffusion or chemical reaction control [33]. The Elovich kinetic model is expressed in Equation (5):

(5) $$q_{t}=\frac{1}{\beta }\ln(\alpha \beta )$$

where *α* is the original adsorption rate constant and *β* is the desorption coefficient, which is related to the coverage of the adsorbate on the surface of the adsorbent and the activation energy for chemical adsorption. The experimental data were fitted to three models using nonlinear least squares through the SOLVER tool, based on the generalized reduced gradient iteration method available in Microsoft Excel (Microsoft 2019, version 1808, Microsoft Corp., Redmond, WA, USA).

Figure 6 shows the nonlinear fitting curves using the expressions of the pseudo-first- and pseudo-second-order kinetic models as solid and dashed lines, respectively. The fitting curve of the Elovich kinetic model is shown in Figure S3. Table 2 summarizes the parameters derived from the three kinetic models. The pseudo-second-order model exhibited the highest correlation coefficients (*R*^2^) among the models, indicating superior alignment with the experimental data in comparison to the pseudo-first-order and Elovich models. Furthermore, the theoretical *q_e_* value calculated from the pseudo-second-order model closely matched the experimental *q_e_*, reinforcing that the pseudo-second-order adsorption mechanism was predominant. Thus, the adsorption of Cu(II) onto EDNFBC suggested that chemisorption was the rate-controlling mechanism. These results indicated that a covalent bond was formed through the sharing of electrons between the metal ions and the adsorbent.

#### 2.4.3. Adsorption Isotherms

Adsorption isotherms are critical for understanding the interactions between the adsorbate and adsorbent, as well as for optimizing the adsorbent use. Several models have been developed to describe liquid–phase adsorption equilibrium data, while the adsorption isotherms of Cu(II) and EDNFBC were analyzed using the Langmuir, Freundlich, and Brunauer–Emmett–Teller (BET) models, which are widely used adsorption isotherm models.

The Langmuir isotherm assumes that adsorption occurs on a homogeneous surface with evenly distributed energy sites on the adsorbent. It assumes that each adsorbate molecule occupies a single site, and no further adsorption occurs once the site is filled, leading to the formation of an adsorbate monolayer on the adsorption surface. The adsorbent surface has sites of identical energy, and each adsorbate molecule is located at a single site, predicting the formation of an adsorbate monolayer on the adsorption surface. Once a site is occupied, no further adsorption occurs, resulting in the surface saturation corresponding to the maximum adsorption capacity [34]. The Langmuir model is expressed in Equation (6).

(6) $$q_{e}=\frac{Q_{m}K_{L}C_{e}}{1+bC_{e}}$$

where *q_e_* is the adsorption capacity of the EDNFBC at equilibrium, *Q_m_* is the maximum monolayer adsorption capacity, *K_L_* is the Langmuir constant, and *C_e_* is the equilibrium concentration of Cu(II). The Langmuir isotherm also predicts adsorption behavior using the dimensionless separation factor, *R_L_*, as shown in Equation (7).

(7) $$R_{L}=\frac{1}{{1+K}_{L}C_{0}}$$

where *C*_0_ denotes the initial metal concentration. This parameter indicates whether adsorption is irreversible (*R_L_* = 0), favorable (0 < *R_L_* < 1), linear (*R_L_* = 1), or unfavorable (*R_L_* > 1) [35].

The Freundlich isotherm is an empirical model describing non-ideal and reversible adsorption. This isotherm is widely recognized for its applicability to multisite adsorption processes [36]. The Freundlich model is expressed in Equation (8) as follows:

(8) $$q_{e}=K_{F}{C_{e}}^{1/n}$$

where *K_F_* is the Freundlich constant corresponding to the maximum adsorption capacity and *n*^−1^ is a dimensionless constant. *n*^−1^ is an empirical parameter related to the isotherm shape; the adsorption process can be classified as irreversible (*n*^−1^ = 0), favorable (0 < *n*^−1^ < 1), or unfavorable (*n*^−1^ > 1) [37].

Finally, the Brunauer–Emmett–Teller (BET) isotherm model can be used to describe a multilayer adsorption system [38] and is expressed in Equation (9) as follows:

(9) $$q_{e}=\frac{Q_{mBET}K_{b}C_{e}}{(C_{0}-C_{e})\left[1+\left(K_{b}-1\right)\frac{C_{e}}{C_{0}}\right]}$$

where *Q_mBET_* is the maximum monolayer adsorption amount and *K_b_* is a constant representing the energy of interaction with the surface.

Based on the experimental results, the adsorption capacity at equilibrium (*q_e_*) was calculated using Equation (10) as follows:

(10) $$q_{e}=\frac{(C_{0}-C_{e})V}{m}$$

where *C*_0_ (mg/L) is the initial concentration of Cu(II), *C_e_* is the concentration of Cu(II) at equilibrium, *V* (L) is the volume of the Cu(II) solution, and *m* (g) is the weight of EDNFBC. The experimental data were fitted to three isotherm models using the SOLVER tool in Microsoft Excel.

**Figure 7:**
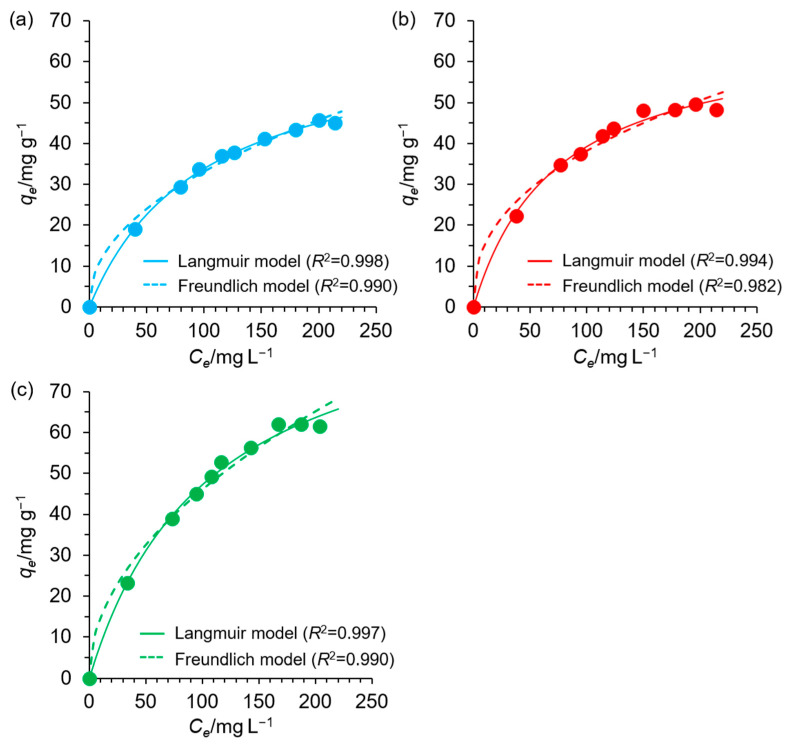
Effect of the equilibrium concentration of Cu(II) on the adsorption capacity at equilibrium, fitted to the Langmuir and Freundlich isotherms for the adsorption of Cu(II) using EDNFBC (**a**) 1, (**b**) 2 and (**c**) 3. The experiments were performed at pH 5 and 298 K using a dose of 0.9 mg/mL EDNFBC. The equilibrium time in the figure was set to 120 min.

Figure 7 shows nonlinear fitting curves for the Langmuir and Freundlich isotherm models, and Figure S4 shows the BET isotherm model. The isotherm parameters are listed in Table 3. Based on the *R*^2^ values, the Langmuir model provided the best fit (*R*^2^ = 0.994–0.998), outperforming the Freundlich and BET models. Describing the adsorption process using the Langmuir isotherm model, it can be stated that Cu(II) adsorption occurred on a homogenous surface, resulting in the formation of a monolayer coverage on which Cu(II) was evenly distributed. The energy associated with adsorption was constant, and no interactions were observed among the Cu(II) adsorbed on the EDNFBC surface. The calculated *R_L_* values within the experimental concentration range were between 0 and 1, indicating that adsorption readily occurred. The theoretical maximum adsorption capacities were calculated to be 67.2, 67.9, and 97.3 mg g^−1^ for EDNFBC 1–3, respectively. Based on the Freundlich isotherm, the estimated *n*^−1^ values laid in the range 0.406–0.503, indicating that the adsorption of Cu(II) onto EDNFBC was favorable according to the Freundlich model. In contrast, the *R*^2^ values for the BET models (0.871–0.951) were significantly lower than those for the Langmuir model. Furthermore, the *Q_mBET_* values were inconsistent with the corresponding experimental *q_e_* values. These results indicated that the Cu(II) adsorption onto EDNFBC was not a multilayer process involving interactions between the Cu(II) adsorbed and the surface of EDNFBC.

From the analysis of the three isotherm models, it can be concluded that the adsorption of metal ions onto EDNFBC followed a Langmuir-type model. This suggests that the adsorbed metal ions do not interact with each other, and only a monolayer is formed at maximum adsorption. The adsorption process was proven not to entail physical adsorption, as discussed in Section 2.4.2. The absence of physisorption was attributed to the dominance of chemisorption, characterized by electron sharing or transfer between the Cu(II) ions and the functional groups on EDNFBC. As presented at the bottom of Figure 1, the adsorption of Cu(II) onto EDNFBC was primarily driven by the chelation effect of the EDTA functional groups with the carboxylic and amine groups serving as coordination sites.

**Figure 8:**
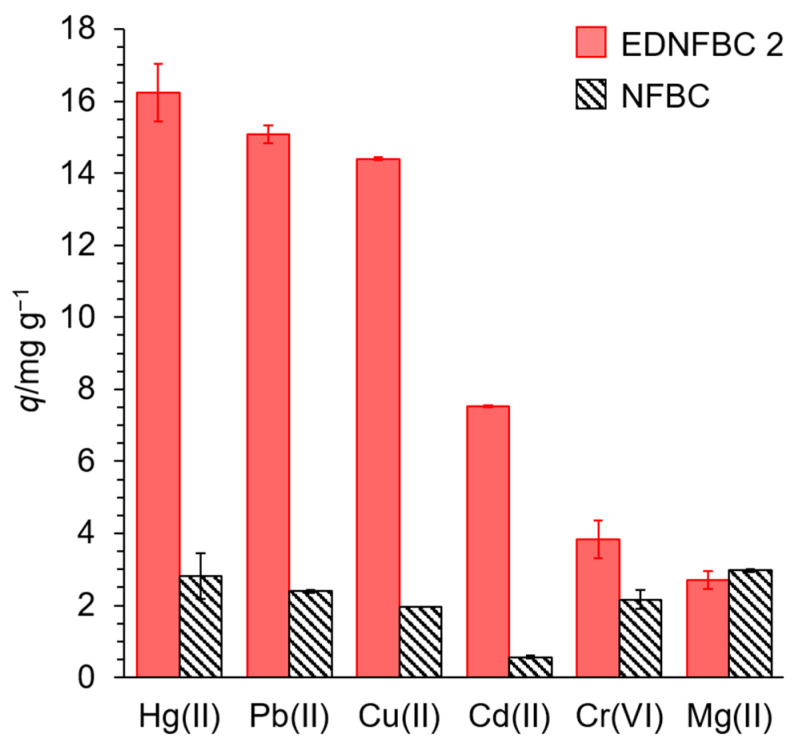
Adsorption of various metal ions onto EDNFBC 2 and NFBC. Initial concentration of metal ions, 25 mg/L; dosage of EDNFBC 2 or NFBC, 0.9 mg/mL; contact time, 120 min; shaking rate, 120 rpm; 298 K.

Next, the amount of EDTA substituted into NFBC (i.e., the degree of substitution = D.S.) was estimated using *Q_m_*. The D.S. was defined as the amount of EDTA substituted per anhydroglucose unit. Cu(II) adsorption on EDNFBC is a chemical adsorption process, indicating that Cu(II) adsorption on EDNFBC occurred mainly via chelation between the EDTA groups and Cu(II). EDTA forms stable chelating complexes with metal ions in a 1:1 ratio, irrespective of the charge of the cation. It has been reported that EDTA substituted on polysaccharides forms a 1:1 ratio chelate with metal ions, even when one out of four carboxyl groups in the EDTA structure are used to bind with the polysaccharides [39]. Thus, the number of moles of Cu(II) adsorbed onto the EDNFBC is equivalent to the number of substituted EDTA molecules. Unmodified NFBC exhibited a low Cu(II) adsorption capacity, as shown in Figure 8. The amount of Cu(II) adsorbed by NFBC can be subtracted from the *Q_m_* of EDNFBC, and the D.S. can subsequently be calculated using Equation (11) as follows:

(11) $$D.S.\;=\;\frac{{162(Q}_{m}-Q_{NFBC})}{64-340(Q_{m}-Q_{NFBC})}$$

where *Q_NFBC_* is the Cu(II) adsorption capacity of NFBC, 64 and 162 are the molar masses of the copper and anhydroglucose units, respectively, and 340 is the increase in molecular mass due to EDTA substitution. The determined D.S. for each EDNFBC type is listed in Table 1. The results show that the D.S. increased with an increase in the feed ratio of EDTAM and NFBC during EDNFBC synthesis, suggesting that D.S. can be controlled using the feed ratio of EDTAM and NFBC.

#### 2.4.4. Adsorption for Various Metal Ions

The adsorption of various metal ions is crucial for practical industrial applications. To investigate the adsorption ability of EDNFBC for various metal ions, an adsorption test was carried out using metal ions such as Pb(II), Cd(II), Cr(VI), Hg(II), and Mg(II). Experiments were performed using a solution containing 25 mg/L of each metal ion. In addition, adsorption tests were conducted on the raw NFBC material. Figure 8 shows the adsorption of various metal ions onto EDNFBC 2 and NFBC. The raw NFBC material exhibited slight adsorption of all metal ions. It is hypothesized that this phenomenon is due to the carboxyl groups of CMC that remain on the surface of NFBC, forming chelates with metal ions, thereby facilitating the adsorption of each metal.

EDNFBC demonstrated higher adsorption capacities for Hg(II), Pb(II), and Cu(II) at 16, 15, and 14 mg g^−1^, respectively, than those of NFBC. However, the amount of Cd(II) adsorbed was lower than that of the other three metals, yet it was higher than that of NFBC. For Cr(VI), the adsorption capacity of EDNFBC was only slightly improved, with a value of 3.8 mg g^−1^ compared to 2.2 mg g^−1^ for NFBC. This slight enhancement in adsorption can be attributed to the presence of Cr(VI) as an oxoanion. At pH 5.0, Cr(VI) predominantly exists as Cr_2_O_7_^2−^ and HCrO_4_^−^ [40]. Therefore, the formation of a chelate complex between Cr(VI) and EDTA was unlikely. Instead, it is hypothesized that the adsorption behavior was more likely influenced by electrostatic interactions between the protonated amino groups of EDTA and the Cr(VI) species.

Conversely, the amount of adsorbed Mg(II) by EDNFBC was slightly lower than that by NFBC. EDTA has a high chelation ability for transition metals such as Cu(II), Pb(II), Hg(II), and Cd(II) but a low chelation ability for alkaline earth metals such as Mg (II) [41]. Therefore, it has been demonstrated that metal adsorption by EDNFBC depends on the chelating capacity of EDTA. It is anticipated that EDNFBC will demonstrate high adsorption of transition metal ions in addition to the metal ions examined in this study. In addition, the notable disparity in the adsorption of transition metal ions and alkaline earth metals in EDNFBC indicates that they can be utilized for the selective removal of transition metal ions in the presence of numerous metal ions.

### 2.5. Reusability

**Figure 9:**
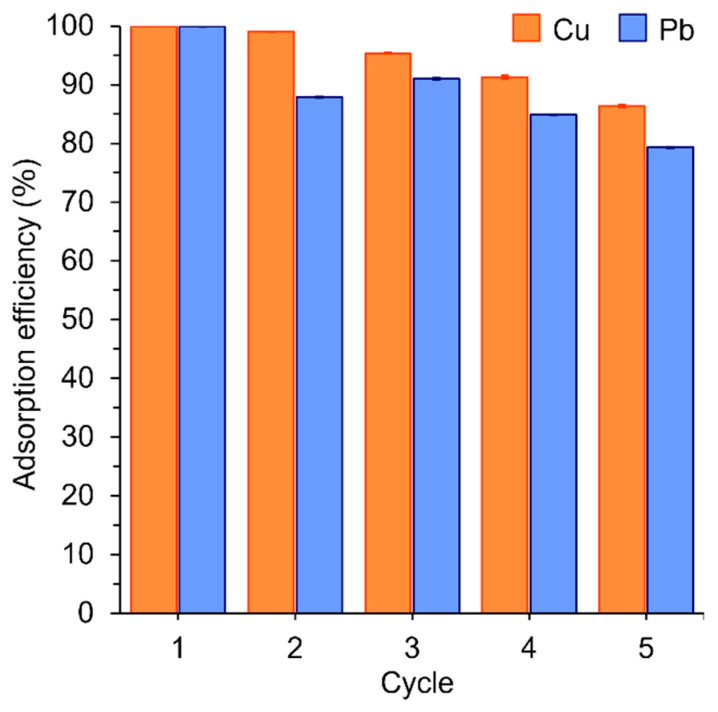
The recyclability of EDNFBC 2 for the adsorption of Cu(II) and Pb(II) after *n* (=1–5) adsorption and desorption cycles. The adsorption process involved an initial metal ion concentration of 25 mg/L, an EDNFBC 2 dosage of 0.9 mg/mL, a contact time of 120 min, a shaking rate of 120 rpm, and a temperature of 298 K. The desorption process involved using 1 mol/L HCl as an eluting solution and an immersion time of 20 min.

Recycling the adsorbent is essential to minimize operating costs. Hence, the used EDNFBC was subjected to five consecutive adsorption–desorption cycles to study its regeneration ability. Normally, desorption is performed using acidic aqueous solutions such as HNO_3_, H_2_SO_4_, and HCl as eluting agents [42,43]. In this study, 1 mol/L HCl was chosen due to its high desorption efficiency and widespread application. First, EDNFBC was used to absorb 25 mg/L Cu(II) or Pb(II) solution at pH 5.0. The adsorbed EDNFBC was then immersed in 1 mol/L HCl as an eluting agent to desorb Cu(II) and Pb(II). The acidic pH protonated the EDTA groups on the EDNFBC. H^+^ ions then competed with metal ions in the adsorbent to bind to the active sites, which desorbed the metal ions from EDNFBC. The EDNFBC was subsequently rinsed with water five times until a neutral pH was achieved. The adsorption efficiency was then calculated after each cycle. Finally, the adsorption efficiency was expressed as the ratio of the amount adsorbed in each cycle to that in the first cycle. For Pb(II), the adsorption efficiency in cycle 2 was lower than in the first cycle, reaching 88% after the first desorption process (Figure 9). However, a slight decrease in the adsorption efficiency was observed for Cu(II), which still exhibited a high value of 99%. The adsorption efficiency gradually decreased with the number of cycles but remained at 86% for Cu(II) and 80% for Pb(II) after five cycles. The adsorption efficiency of Cu(II) was slightly higher than that of Pb(II), possibly because the binding strength between Pb(II) and EDNFBC was higher than that of Cu(II). No significant loss of Cu(II) and Pb(II) adsorption performance was detected even after five cycles, suggesting that the use of EDNFBC for water purification results in reduced operating costs.

EDNFBC offers distinct advantages over conventional industrial sorbents, such as clays, zeolites, and agricultural residues [44,45], particularly in terms of its exceptional reusability. Conventional adsorbents frequently exhibit a decline in adsorption capacity after repeated regeneration cycles due to structural degradation or the loss of active sites. In contrast, EDNFBC demonstrates the capacity to maintain its structural integrity and adsorption efficiency across multiple cycles. This durability allows for repeated use without significant performance loss, making EDNFBC ideal for applications requiring long-term efficiency and high reliability. Additionally, its reusability significantly minimizes waste generation, thereby promoting sustainable strategies for heavy metal remediation.

## 3. Conclusions

In this study, EDTA grafting was performed on an NFBC surface using EDTA monoanhydride to convert NFBC into an efficient metal adsorbent. The EDTA-grafted NFBC remained structurally unchanged despite reacting with EDTA, indicating that the surface hydroxyl groups were selectively substituted by EDTA groups. The adsorption behavior of the EDTA-grafted NFBC for Cu(II) was consistent with the Langmuir isotherm and pseudo-second-order kinetic models, indicating that its adsorption mechanism mainly involved single-molecular layer chemisorption. The EDTA-grafted NFBC demonstrated a low adsorption capacity for alkaline earth metals, such as Mg(II), while exhibiting a high adsorption capacity for transition metals, including Cu(II), Hg(II), Pb(II), and Cd(II). Cr(VI), which predominantly exists as an oxoanion in aqueous solutions, also exhibited low adsorption, indicating the selective adsorption behavior of EDTA-grafted NFBC toward specific metal ions. Moreover, the adsorption efficiency demonstrated notable stability, maintaining consistent levels even after five consecutive adsorption–desorption cycles, indicating its remarkable resilience. Previous studies on cellulose functionalization with EDTA have primarily utilized materials such as fabrics or filters as the raw substrate, which inherently limits their range of applications. In contrast, cellulose nanofibers can be formed into various shapes, including filters and aerogels, offering greater versatility [46,47]. The material developed in this study is also expected to be molded into different shapes depending on the intended application, thereby expanding its potential range of applications. These findings indicate that EDTA-grafted NFBC has considerable potential for use in wastewater remediation and the treatment of heavy metal ion contamination.

## Figures and Tables

**Table 1:** The initial feed amounts of NFBC and EDTA monoanhydride (EDTAM) to prepare suspensions EDNFBC 1–3, and the degree of substitution (D.S.) of each suspension.

Sample	Initial Feed Amount		Feed Molar Ratio of EDTAM/AGU	D.S. ^b^
	NFBC (g) (AGU ^a^ (mmol))	EDTAM (g) (mmol)		
EDNFBC 1	1.00 (6.2)	0.7 (3.1)	0.5	0.25
EDNFBC 2	1.00 (6.2)	1.4 (6.2)	1.0	0.33
EDNFBC 3	1.00 (6.2)	2.9(12.4)	2.0	0.49

^a^ AGU is the number of moles of the anhydroglucose units of NFBC. ^b^ The D.S. was determined based on the maximum amount of Cu(II) ions adsorbed (Section 2.4.3).

**Table 2:** Kinetic parameters for the adsorption of Cu(II) with EDNFBC 2 at pH 5.

Experimental *q_e_* (mg g^−1^)	38.1	
Pseudo-first-order model	*k*_1_ (min^−1^)	0.13
	Calculated *q_e_* (mg g^−1^)	36.1
	*R* ^2^	0.672
Pseudo-second-order model	*k*_2_ (g mg^−1^ min^−1^)	0.0066
	Calculated *q_e_* (mg g^−1^)	37.7
	*R* ^2^	0.940
Elovich model	*α* (mg g^−1^ min^−1^)	1.02 × 10^4^
	*β* (g mg^−1^)	0.36
	*R* ^2^	0.915

**Table 3:** Parameters of the nonlinear Langmuir, Freundlich, and BET isotherms for the Cu(II) adsorption on EDNFBC 1–3.

EDNFBC		1	2	3
Experimental *q_e_* (mg g^−1^) ^a^		45.1	48.3	61.6
Langmuir	*Q_m_* (mg g^−1^)	67.2	78.6	97.3
	*K_L_* (L mg^−1^)	0.010	0.010	0.0094
	*R_L_*	0.50–0.29	0.51–0.30	0.53–0.31
	*R* ^2^	0.998	0.982	0.997
Freundlich	*n* ^−1^	0.467	0.406	0.503
	*K_F_* (L mg^−1^)	3.84	5.89	4.55
	*R* ^2^	0.990	0.982	0.990
BET	*Q_mBET_* (mg g^−1^)	7.23	8.60	13.0
	*K_b_*	8.60 × 10^7^	1.89 × 10^5^	19.1
	*R* ^2^	0.871	0.876	0.951

^a^ *q_e_* of 120 min when *C*_0_ was set to 240 mg/L.

## Data Availability

The original contributions presented in this study are included in the article/Supplementary Materials. Further inquiries can be directed to the corresponding authors.

## Supplementary Materials

The following supporting information can be downloaded at: https://www.mdpi.com/article/10.3390/ma18020374/s1, Materials and Methods; Results and discussion for thermogravimetry (TG)/differential thermal analysis (DTA) of EDNFBC; Figure S1: (a) TG and DTA curves for NFBC and EDNFBC 1–3; Figure S2: (a) Initial (Ti) and (b) final (Tf) degradation temperatures for the second degradation of EDNFBC 1–3 and NFBC as determined using thermogravimetric analysis (Figure S1); Figure S3: Effect of contact time on the adsorption of Cu(II) onto EDNFBC 2 as fitted to the Elovich kinetic model; Figure S4: Effect of the equilibrium concentration of Cu(II) on adsorption capacity at equilibrium fitted to the BET isotherm for the adsorption of Cu(II) using EDNFBC 1–3. References [23,48,49,50,51,52,53] are cited in the Supplementary Materials.
