# Supplementary material for: Nano-Fibrillated Bacterial Cellulose Nanofiber Surface Modification with EDTA for the Effective Removal of Heavy Metal Ions in Aqueous Solutions
Source: Materials (Basel). 2025 Jan 15;18(2):374. doi: 10.3390/ma18020374 (PMC11767098; doi:10.3390/ma18020374)
Supplement: Supplementary file 1 [file materials-18-00374-s001.zip › materials-3388872-supplementary.pdf]

materials MDPI

Supporting Information

Nano-Fibrillated Bacterial Cellulose Nanofiber Surface Modification with EDTA for the Effective Removal of Heavy Metal Ions in Aqueous Solutions

Sayaka Fujita[1,*], Ryosui Sasa[2], Nanami Kinoshita[1], Ryota Kishimoto[1] and Hiroyuki Kono[1,*]

[1] Division of Applied Chemistry and Biochemistry, National Institute of Technology, Tomakomai College, Nishikioka 443, Tomakomai, Hokkaido 059-1275, Japan
[2] Graduate School of Environmental Science, Hokkaido University, N10W5, Kita-ku, Sapporo, Hokkaido 060-0810, Japan

*Corresponding authors: fujita@tomakomai-ct.ac.jp (S. F.); kono@tomakomai-ct.ac.jp (H. K.)
Tel.: +81-144-67-8038 (S.F.); +81-144-67-8036 (H.K.)

1. Materials and Methods

1.1. Materials

A 1.0% (w/v) NFBC suspension in water was prepared by Kusano Sakko, Inc. (Hokkaido, Japan). Prior to use, the precise concentration of the NFBC suspension was determined by measuring the dry weight of 10 mL of the NFBC suspension with an MS-70 infrared moisture meter (A&D Co., Tokyo, Japan). EDTA was purchased from Dojindo Laboratories Co. Ltd. (Kumamoto, Japan). EDTA monoanhydride (EDTAM) was prepared following a method described in a previous study [23]. All other reagents and solvents were of analytical grade and purchased from Fujifilm Wako Pure Chemical Co., Ltd. (Osaka, Japan).

1.2. Preparation of EDNFBC

NFBC suspension (100 mL, dry weight: 1 g; 6.2 mmol of anhydroglucose units [AGU]) was concentrated via centrifugation at 10,000 ×*g* for 20 min to remove as much water as possible. The concentrated NFBC slurry were suspended in 50 mL DMSO. EDTAM (0.7 g, 3.1 mmol, 0.5 molar equivalents to AGU) and DMAP (0.4 g, 3.1 mmol, molar equivalent to the EDTAM) were then added to the NFBC suspension in DMSO. Next, the mixture was vigorously stirred at 298 K for 24 h. After this period, the the product was separated by centrifugation at 10,000 ×*g* for 20 min and washed three times with deionized water. The mixture was then dialyzed using a dialysis membrane tube (Thermo Fisher Scientific, Waltham, MA, USA) with a molecular weight cutoff of 12,000 kDa against deionized water to remove the unreacted reagents. Finally, a 1 wt% EDNFBC 1 suspension in water was obtained. The suspensions EDNFBC 2 and 3 were similarly prepared by changing the amount of EDTAM to 1.4 g (6.2 mmol) and 2.9 g (12.4 mmol), respectively (Table 1).

1.3. Structural Characterization of EDNFBC

FTIR spectra were acquired using an ALPHA II FTIR spectrometer (Bruker Optics GmbH & Co., KG, Karlsruhe, Germany) at 295 K. The samples were finely ground, mixed with potassium bromide (KBr) powder, and pressed into transparent pellets. Spectral data were scanned from the wavenumber range 4000 to 500 cm^{-1} with a resolution of 4 cm^{-1},

averaging 32 scans per measurement. Background spectra, recorded under identical conditions, were subtracted to enhance accuracy.

X-ray diffraction (XRD) patterns for freeze-dried NFBC and EDNFBC 1–3 were measured using a Bruker D8 Advance diffractometer (Bruker AXS, Karlsruhe, Germany). The system utilized Ni-filtered CuKα (λ = 1.54056 Å) operating at 40 kV and 30 mA. Scans were performed in the 2θ range of 5–30° with scan speed of 1.000°/min. The crystallinity index (C.I.) of the samples was determined by calculating the ratio of the crystalline cellulose peak areas to the total peak areas, including both the crystalline and amorphous cellulose peaks. Nonlinear least-squares fitting of the diffractograms was carried out using TOPAS software (version 4.2, Bruker AXS, Germany).

1.4. Thermogravimetry (TG)/Differential Thermal Analysis (DTA)

Thermal properties of the samples were assessed usng EVO2 TG 8120 Plus thermogravimetric dynamic thermal analyzer (Rigaku Co., Tokyo, Japan). Approximately 15 mg of each sample was placed in a platinum crucible and heated from 30 °C to 700 °C at a constant heating rate of 5 °C min^{-1} under a continuous nitrogen gas flow.

1.5. Morphological Characterization

Morphological observations of the freeze-dried NFBC and EDNFBC samples were performed using field-emission scanning electron microscopy (SEM; JSM-7500F, JEOL Ltd., Tokyo, Japan). Samples were mounted on aluminium grids with carbon tape and coated with a platinum layer of 4 nm thick. Obsevations were performed at an acceleration voltage of 5 kV. SEM images were processed using the ImageJ (version 1.53 m, the U.S. National Institutes of Health, MD, USA) to calculate the fiber diameters. Average widths and standard deviations were determined based on measurements of 50 randomly selected fibers.

1.6. Adsorption Experiments

1.6.1. Effect of pH on Adsorption

The influence of pH on Cu(II) adsorption was examined using a batch process. Briefly, a Cu(II) stock solution (1000 mg/L) prepared from copper (II) nitrate trihydrate and 0.1 mol/L HNO_3, was diluted with deionized water to yield a 27.5 mg/L solution of Cu(II). The pH of this solution was adjusted to 2–5 by adding 1 mol/L HNO_3 or NaOH. This Cu(II) solution (5 mL) was poured into a centrifuge tube, and 1 wt% EDNFBC (0.5 mL) was added. The initial concentrations of Cu(II) and EDNFBC were 25 mg/L and 0.9 mg/mL, respectively. The mixture was immediately shaken at 150 rpm at 298 K for 120 min, and then the suspension was centrifuged at 10,000 ×*g* for 3 min. The residual Cu(II) concentration in the supernatant was determined using a 200 Series AA atomic absorption spectrometer (Agilent Technologies Inc., CA, USA) with a flame atomizer (air/acetylene) and a hollow cathode lamp (wavelength of 324.7 nm). All experiments were conducted in triplicate, and the results were averaged.

1.6.2. Adsorption Kinetics Study

Briefly, the Cu(II) solution (110 mg/L, pH 5, 20 mL) was poured into a centrifuge tube, and 2 mL of 1 wt% EDNFBC was added. The initial concentrations of Cu(II) and EDNFBC were 100 mg/L and 0.9 mg/mL, respectively. The mixture was shaken at 150 rpm and 298 K for predefined times, and 2 mL was removed from the mixture. The suspension was centrifuged at 10,000 ×*g* for 3 min, and the residual Cu(II) concentration in the supernatant was determined. The experimental data were fitted to the selected models using nonlinear least squares through the SOLVER tool, based on the generalized reduced gradient iteration method available in Microsoft Excel.

1.6.3. Adsorption Isotherm

To study the adsorption isotherm of NFBC, adsorption was performed at Cu (II) concentrations ranging from 50–240 mg/L (pH 5.0). Except for the difference in the concentration of Cu(II), adsorption was also carried out under the same conditions as those described in Section 4.4.1. The experimental data were fitted to the Langmuir, Freundlich, and Brunauer–Emmett–Teller (BET) isotherm models using the SOLVER tool in Microsoft Excel.

1.6.4. Adsorption for Various Metal Ions

Herein, adsorption was performed using Pb(II), Cd(II), Cr(VI), Hg(II) and Mg(II) ions instead of Cu(II). Briefly, metal solutions were prepared using lead nitrate, cadmium nitrate, potassium dichromate, mercury chloride, and magnesium chloride. Except for the difference in metal species, adsorption was carried out under the same conditions described in Section 2.6.1. The initial concentration of metal ions was 25 mg/L, the concentration of EDNFBC was 0.9 mg/mL, and the pH was 5.0. The concentrations of Pb(II), Cd(II), Cr(VI), Hg(II) and Mg(II) were determined using atomic absorption spectrometry at wavelengths of 217.0, 228.8, 357.9, 253.7 and 285.2 nm, respectively. For comparison purposes, an adsorption experiment was conducted using NFBC instead of EDNFBC.

1.7. Desorption and Reusability

After the adsorption of Cu(II) or Pb(II) as described in 2.6.4, EDNFBC was collected by filtration using an ultrafiltration tube (Vivaspin 6, Sartorius Stedim Biotech GmbH, Germany) with a molecular weight cutoff of 10,000, and then immersed in a 1.0 mol/L aqueous HCl solution for 20 min to remove the metal ions. The regenerated EDNFBC was then washed with pure water to achieve neutrality, undergoing five consecutive cycles of adsorption and desorption. The adsorption capacity of the regenerated EDNFBC after *n* cycles was determined using the aforementioned protocol.

2. Thermogravimetry (TG)/Differential Thermal Analysis (DTA) of EDNFBC

Figures S1 depicts the TG/DTA curves of NFBC and EDNFBC 1–3. The TG curve of NFBC revealed there distinct stages of weight loss. In the first stage, approximately 5 wt% of weight loss occurred below 110 °C, which is attributed to the vaporization of physically adsorbed water and intermolecular hydrogen-bonded water from cellulose chains [48]. The second stage of weight reduction, observed between 220 °C and 290 °C, corresponds to the depolymerization of polymer chains and the cleavage of glycosidic linkages [49]. The third stage, occurring above 370 °C, associated with the thermal decomposition of pyranose rings and the breakdown of residual carbon [50]. The TG curves of EDNFBC displayed a similar three-stage weight loss pattern as observed for NFBC.

Figures S2 shows the initial and final stages of the second thermal decomposition, along with the corresponding the initial (T_i) and final (T_f) decomposition temperatures of NFBC and EDNFBC 1–3. The T_i and T_f values of the NFBC were 271 and 346 °C, respectively. The T_i and T_f values of EDNFBC 1–3 were slightly lower than those of NFBC. In general, the introduction of functional groups into polysaccharides disrupts intermolecular interaction, resulting in decreased the thermal stability [51,52]. The decrease in the T_i values of the EDNFBC was attributed to the introduction of EDTA onto the fiber surface of NFBC. DTA showed the main degradation peak for NFBC at 310 °C. EDNFBC showed degradation peaks at approximately 290 °C and 370 °C; these were attributed to the degradation of the substituted EDTA [53].

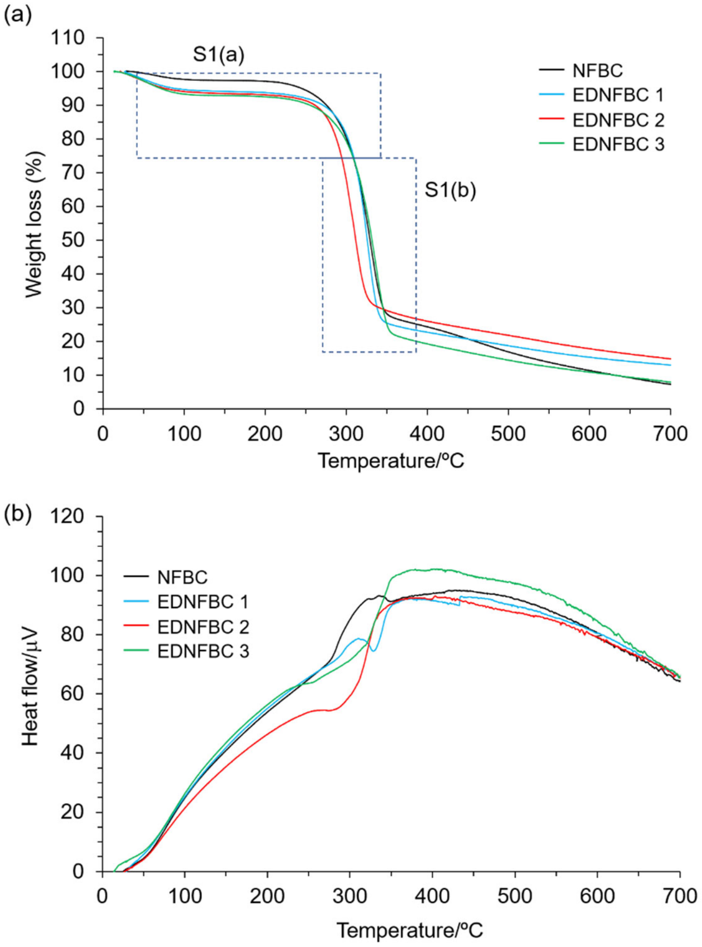

Figure S1. (a) Thermogravimetric (TG) and (b) differential thermogravimetric analysis curves for NFBC and EDNFBC 1–3. The detailed TG curves, which are highlighted by the dashed lines, are shown in Figures S2(a) and (b).

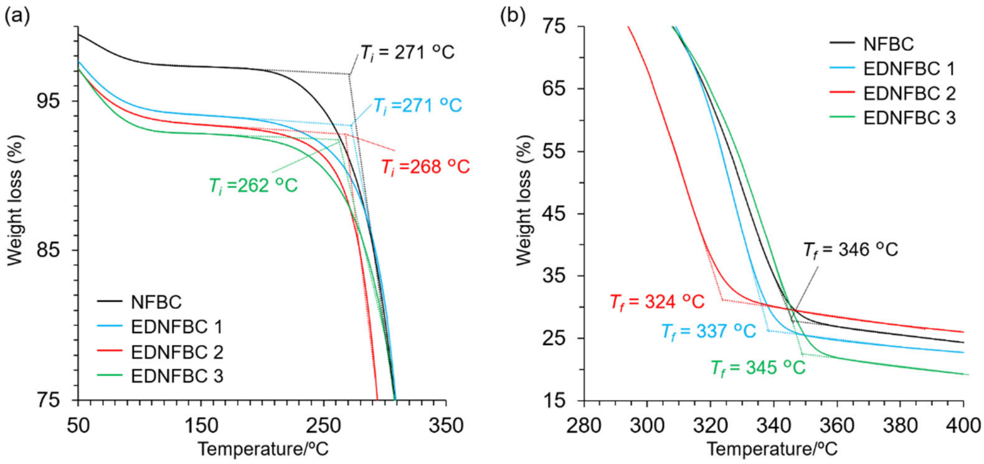

Figure S2. (a) Initial (T_i) and (b) final (T_f) degradation temperatures for the second degradation of EDNFBC 1–3 and NFBC, as determined via thermogravimetric analysis.

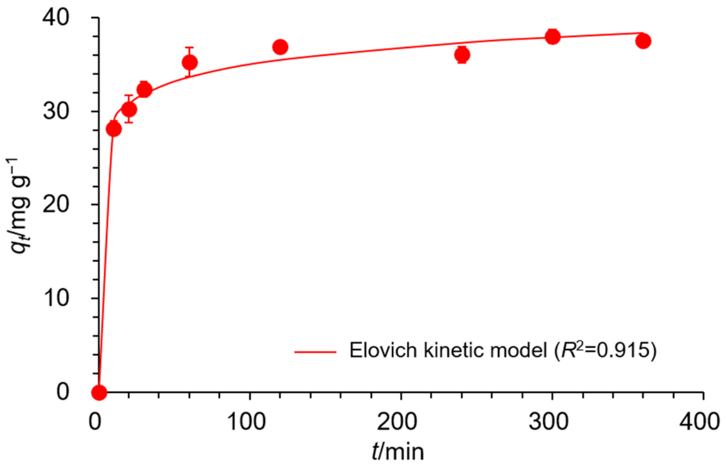

Figure S3. Effect of contact time on the adsorption of Cu(II) on EDNFBC 2 as fitted to the Elovich kinetic model.

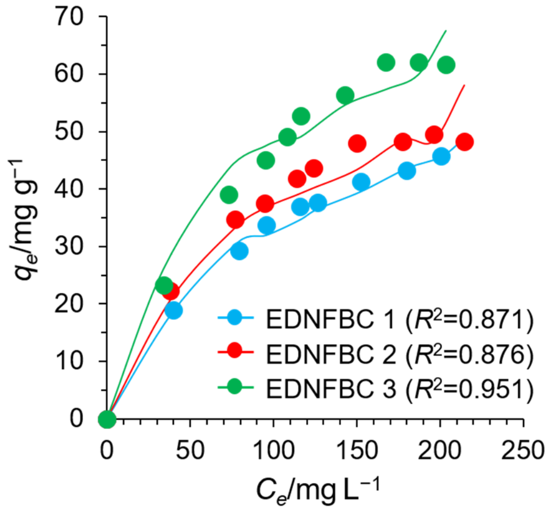

Figure S4. Effect of the equilibrium concentration of Cu(II) on adsorption capacity at equilibrium a fitted to the BET isotherm for the adsorption of Cu(II) using EDNFBC 1–3.
